# Coexisting Ulnar Nerve Schwannoma and Lateral Humeral Condyle Nonunion Presenting as Tardy Ulnar Nerve Palsy: A Report of a Rare Case

**DOI:** 10.7759/cureus.81015

**Published:** 2025-03-22

**Authors:** Pankaj Kabra, Mende Vikram Kumar Yadav, Anil Kumar Chitumalla

**Affiliations:** 1 Department of Orthopaedics, Nizam's Institute of Medical Sciences, Hyderabad, IND; 2 Department of Orthopaedics, Kamineni Institute of Medical Sciences, Narketpally, IND

**Keywords:** cubital tunnel syndrome, lateral humeral condyle nonunion, neuropathy, schwannoma, ulnar nerve

## Abstract

Tardy ulnar nerve palsy (TUNP) is a progressive neuropathy commonly associated with cubitus valgus deformity following lateral humeral condyle nonunion. However, concurrent ulnar nerve schwannoma as a contributing factor remains undocumented. We present a rare case in which both chronic traction neuropathy and intraneural compression coexisted, with the schwannoma remaining undetected on high-resolution ultrasound (HRUS) preoperatively.

A 44-year-old male professional driver presented with a six-month history of progressive ulnar neuropathy, characterized by paresthesia, intrinsic muscle weakness, and occupational impairment. Preoperative evaluation revealed severe functional limitation, with a DASH (Disabilities of the Arm, Shoulder, and Hand) score of 72 and grip strength of 40 kg compared to 70 kg contralaterally. Electrodiagnostic studies confirmed ulnar neuropathy with axonal damage. While HRUS detected nerve compression, it failed to reveal a 0.8 cm schwannoma, which was only identified intraoperatively. The patient underwent microsurgical schwannoma excision, anterior subcutaneous transposition, and microneurolysis.

Postoperatively, the patient demonstrated progressive neurological recovery, achieving a DASH score of 5, restored grip strength (70 kg bilaterally), and normalized two-point discrimination (3-4 mm) within 24 months. This case highlights the dual mechanisms of ulnar nerve injury, involving both chronic traction from cubitus valgus and focal compression from an occult schwannoma. The false-negative HRUS result underscores the limitations of ultrasonography in detecting small intraneural tumors. Given the intraoperative discovery of an occult schwannoma, this case highlights the potential role of MRI in cases of unexplained or refractory ulnar neuropathy.

These findings reinforce the importance of comprehensive surgical strategies in managing complex peripheral nerve pathologies.

## Introduction

Tardy ulnar nerve palsy (TUNP) is a chronic condition characterized by the delayed onset of ulnar neuropathy. The ulnar nerve is particularly vulnerable due to its anatomical course around the elbow joint, making it susceptible to stretching or irritation when structural alterations occur [1]. This condition is a common late complication of traumatic cubitus valgus deformity, often resulting from nonunion or malunion of a distal lateral condyle fracture in children [2]. Additionally, a malunited pediatric supracondylar fracture can lead to cubitus varus deformity, which may also contribute to the development of TUNP [3].

The ulnar nerve is relatively fixed as it passes behind the medial epicondyle of the humerus. An increase in valgus deformity at the elbow joint can stretch the nerve, leading to neuropraxia, a temporary condition that typically resolves through axonal remyelination within three weeks to three months. However, if the deforming force persists, it may cause axonal injury within the endoneurial tube, resulting in intraneural scarring and fibrosis, ultimately leading to axonotmesis. The prognosis of axonotmesis depends on the extent of intra-neural damage, though neurotmesis is rare in TUNP [4]. Ulnar nerve recovery largely depends on the severity of the injury prior to surgery and the success of deformity correction. Most experts recommend anterior transposition of the ulnar nerve to relieve tension, which may be performed with or without correcting the cubitus valgus deformity [5].

Schwannomas are benign tumors that arise from Schwann cells, which surround peripheral, cranial, and autonomic nerves. Schwannomas of the ulnar nerve can cause compression, disrupt axonal transport, and result in nerve dysfunction. These tumors typically present as firm, oval-shaped masses, usually less than 3 cm in size, with a smooth surface, well-defined capsule, and a yellowish to whitish-gray appearance. Early symptoms often include painless swelling, which may progress to pain, paresthesia, hypoesthesia, and motor deficits. Surgical excision is the primary treatment, with the goal of complete removal while preserving nerve function. Although surgery is recommended for symptomatic cases, it carries a risk of temporary or permanent sensory or motor deficits [6].

Here, we present a rare case of a coexisting schwannoma of the ulnar nerve within the cubital tunnel, associated with nonunion of the lateral humeral condyle, leading to TUNP. The uniqueness of this case lies in the combination of these conditions, where the deformity resulting from the nonunion predisposes the ulnar nerve to both mechanical stretching and compression. The schwannoma, measuring only 0.8 cm and located within the confined space of the cubital tunnel, contributed to a subtle clinical presentation, making its diagnosis challenging. Despite its small size, the tumor played a significant role in the development of neuropathy. The preoperative evaluation focused on TUNP, with anterior transposition planned as the primary surgical approach. However, intraoperative exploration unexpectedly revealed the schwannoma, emphasizing the importance of heightened clinical awareness when managing elbow deformities. This case highlights the critical role of surgical precision and intraoperative vigilance, as anterior transposition alone would have been insufficient. Excision of the schwannoma was essential for optimal functional recovery.

## Case presentation

A 44-year-old, right-hand dominant male professional driver presented with a six-month history of progressive paresthesia and hand weakness, primarily affecting the ulnar nerve distribution of his right upper limb. His primary functional limitation was difficulty gripping small objects, such as car keys, which significantly impaired his ability to work. Symptoms initially manifested as intermittent paresthesia along the medial elbow and forearm, which worsened with prolonged elbow flexion during driving. Over time, the paresthesia became constant, and progressive intrinsic muscle weakness developed. Two weeks prior to presentation, the patient experienced a rapid worsening of symptoms, resulting in persistent numbness, severe grip weakness, and an inability to perform occupational tasks.

The patient’s medical history was significant for a childhood lateral humeral condyle fracture at age eight, which resulted in nonunion and subsequent cubitus valgus deformity. He denied any history of diabetes, thyroid disease, cervical radiculopathy, or autoimmune disorders. He had never undergone prior elbow surgeries.

**Figure 1:**
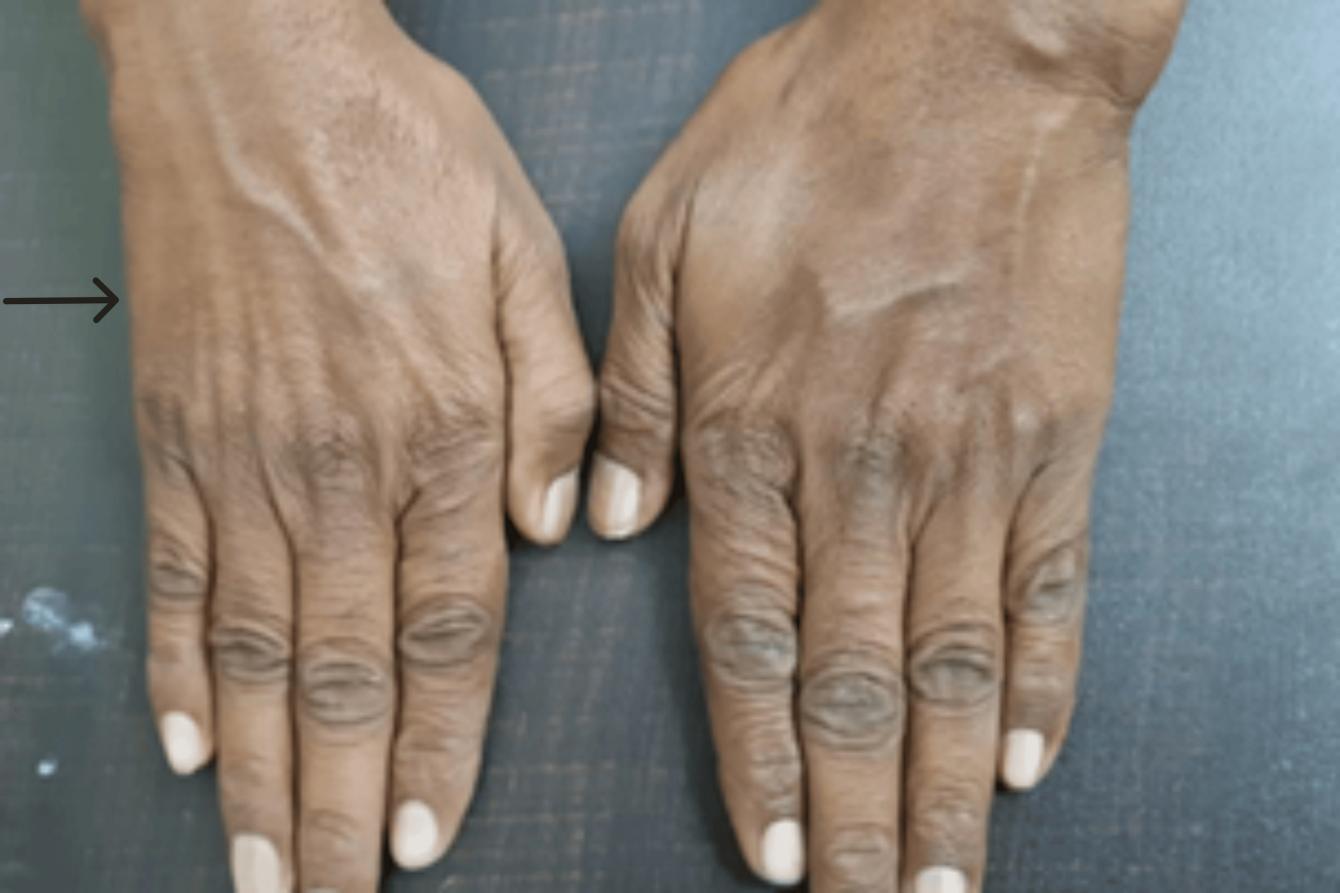
Clinical image demonstrating muscle wasting in the right hand.

On physical examination, the right elbow exhibited cubitus valgus deformity, with a carrying angle of 18° compared to 12° on the contralateral side. Significant muscle wasting was noted in the hypothenar eminence and interossei (Figure 1). Tinel’s sign was positive, and ulnar nerve subluxation was observed during elbow flexion. The active range of motion (ROM) was 15°-110°, with discomfort at the extremes of flexion and extension. Grip strength was 40 kg on the affected side compared to 70 kg contralaterally.

**Figure 2:**
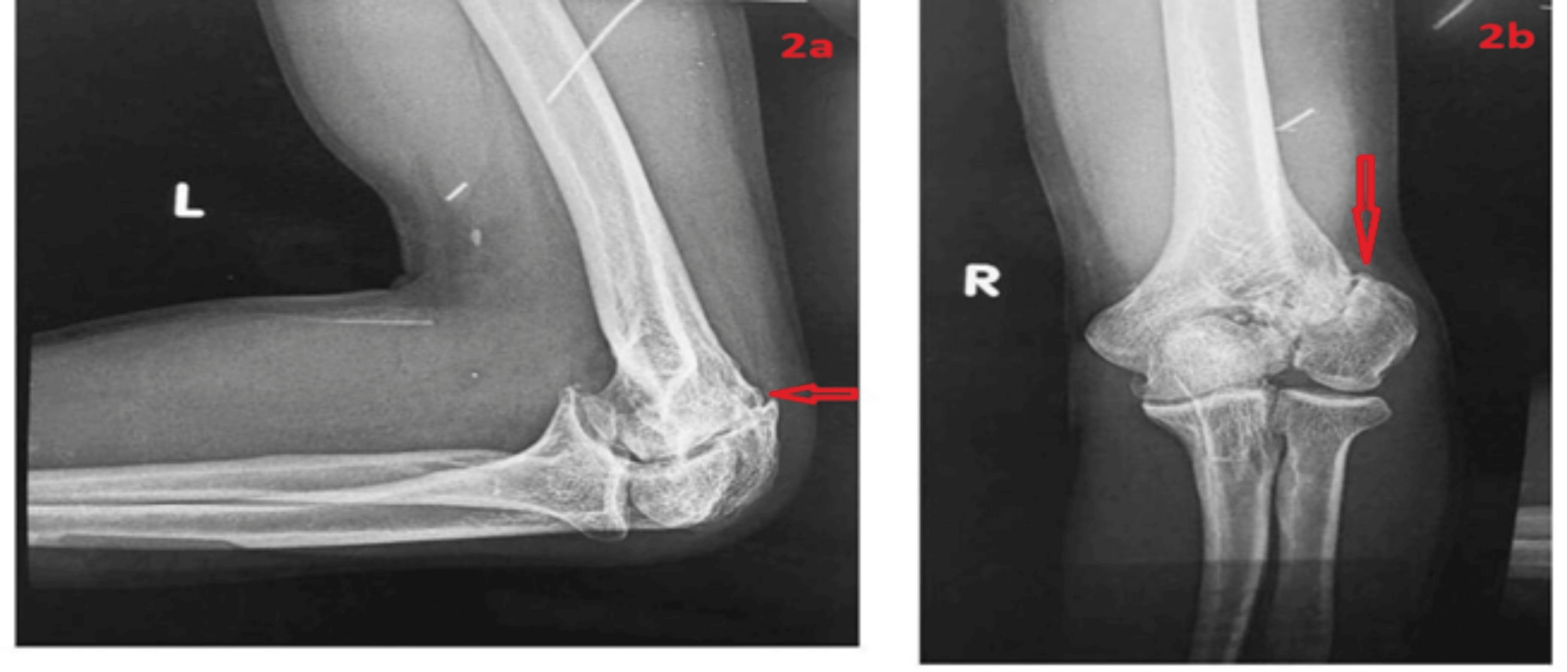
Radiographic evaluation of lateral humeral condyle nonunion. (a) Lateral view showing nonunion of the lateral humeral condyle. (b) Anteroposterior view showing nonunion of the lateral humeral condyle.

A radiographic evaluation confirmed nonunion of the lateral humeral condyle (Figure 2). Preoperative electrodiagnostic studies confirmed severe conduction abnormalities, consistent with axonal dysfunction. Motor latency was prolonged (7.5 ms), conduction velocity was significantly reduced (32 m/s), and compound muscle action potential (CMAP) amplitude was diminished (2.1 mV), indicating axonal degeneration. High-resolution ultrasound (HRUS) revealed nerve compression within the cubital tunnel but failed to identify any mass lesions. MRI was not performed preoperatively, as the initial clinical impression favored isolated TUNP secondary to cubitus valgus deformity, with no overt suspicion of a space-occupying lesion. However, given the intraoperative discovery of a schwannoma, this case highlights the potential diagnostic utility of MRI in identifying occult intraneural tumors when HRUS findings are inconclusive.

Given the longstanding nature of the cubitus valgus deformity, a supracondylar osteotomy was considered. However, because the carrying angle was below the 20° threshold for routine correction, and the patient had functionally adapted over 36 years, osteotomy was deemed unnecessary at this stage. Instead, the primary surgical goal was relieving ulnar nerve compression through anterior transposition.

**Figure 3:**
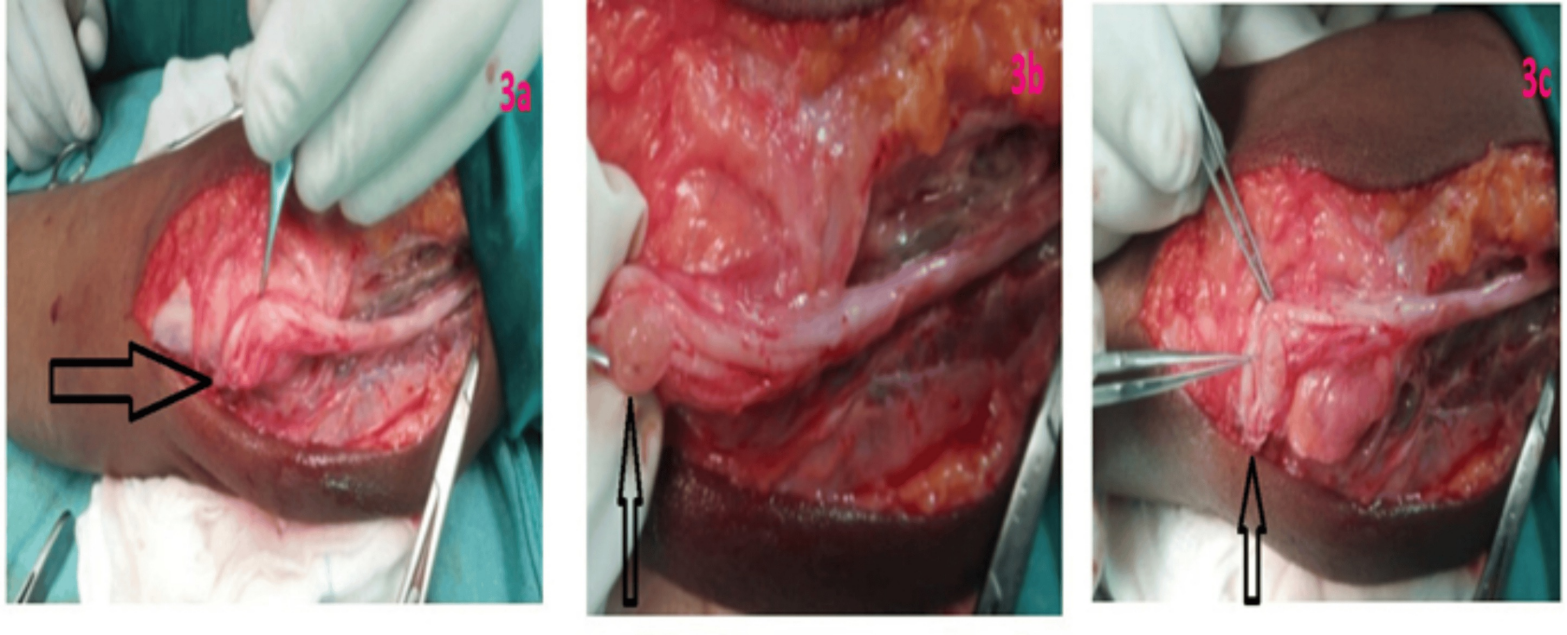
Intraoperative images. (a) Intraoperative image of the tumor within the ulnar nerve. (b) Close-up image of the nerve and tumor. (c) Post-excision image showing the surgical site after tumor removal.

The procedure was performed with the patient in a supine position, with the elbow flexed at 90-110° for optimal exposure of the cubital tunnel. A posteromedial incision was made, and the ulnar nerve was carefully dissected from the medial epicondyle to the proximal forearm under loupe magnification. Continuous intraoperative nerve monitoring was utilized to ensure safe handling. During the procedure, an unexpected finding of a 0.8 cm schwannoma was noted within the ulnar nerve at the cubital tunnel. The tumor was encapsulated and smoothly lobulated, exhibiting classic features of a benign schwannoma. Using microsurgical techniques, the schwannoma was carefully excised, ensuring the preservation of the surrounding nerve fascicles (Figure 3). Histopathological examination confirmed a benign schwannoma, with no evidence of atypia or malignancy in the sections examined.

Following microneurolysis and anterior subcutaneous transposition, the ulnar nerve was placed tension-free in its new position. Final electrophysiological testing confirmed intact motor and sensory function before closure.

Postoperatively, the patient exhibited progressive functional improvement over 24 months. The DASH (Disabilities of the Arm, Shoulder, and Hand) score improved from 72 preoperatively to 5, with incremental gains at three, six, 12, and 18 months. Grip strength was measured using a standardized Jamar dynamometer, following the American Society of Hand Therapists (ASHT) protocol. The patient was positioned in a seated posture, with the elbow flexed at 90°, forearm neutral, and wrist slightly extended (30°). Three consecutive trials were performed for each hand, and the mean value was recorded to minimize variability due to effort or pain. Under these controlled conditions, grip strength demonstrated objective improvement, increasing from 40 kg preoperatively to 70 kg bilaterally by 18 months. Pain levels, assessed via the visual analog scale (VAS), decreased from 6/10 preoperatively to complete resolution by 18 months. Two-point discrimination improved from >10 mm preoperatively to 3-4 mm at 18 months, further validating sensorimotor recovery. Postoperative electrodiagnostic studies at 18 months demonstrated significant objective improvement, reflecting axonal regeneration, and functional nerve recovery. Motor latency decreased from 7.5 ms preoperative to 3.2 ms postoperative, conduction velocity increased from 32 m/s to 54 m/s, and CMAP amplitude improved from 2.1 mV to 6.8 mV.

**Figure 4:**
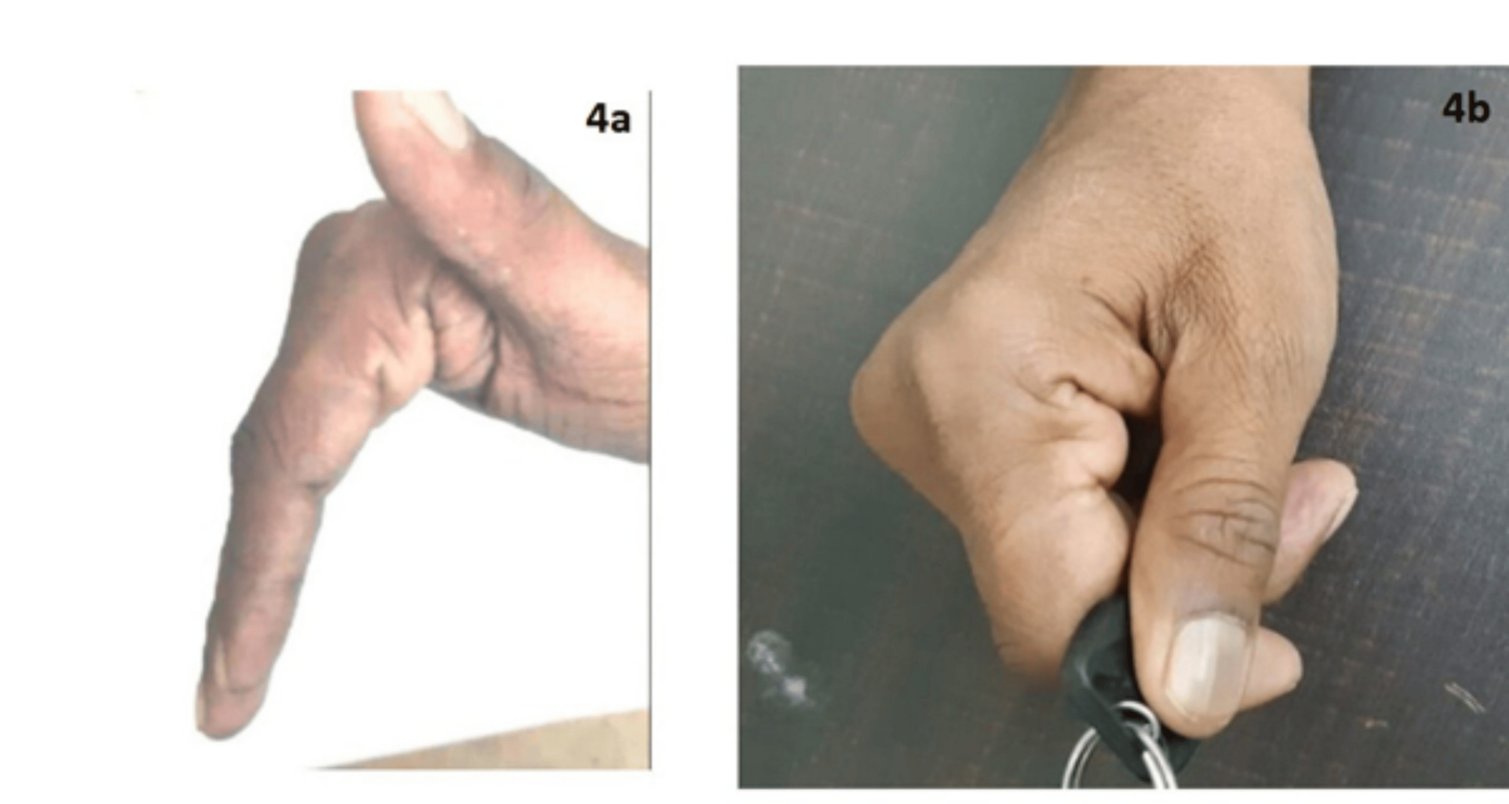
Postoperative functional outcome images. (a) Clinical image showing intrinsic plus hand position after surgery. (b) Postoperative image showing key pinch function after surgery.

By four months, the patient resumed occupational driving with an automatic transmission. By six months, he was able to drive a manual transmission vehicle with intermittent rest breaks. By 12 months, full unrestricted occupational function was restored, with sustained intrinsic muscle recovery confirmed at 24 months (Figure 4).

This case underscores the importance of intraoperative exploration in refractory neuropathies, particularly in patients with concurrent structural deformities. The coexistence of a schwannoma and traction neuropathy presents a complex interplay of nerve compression mechanisms. Traction neuropathy likely played the primary role, but the schwannoma’s location within the cubital tunnel may have contributed to additional compression. The false-negative HRUS finding further highlights the diagnostic limitations of ultrasonography in detecting small intraneural tumors, reinforcing the need for advanced imaging in cases of unexplained neuropathy.

## Discussion

TUNP is associated with various pathological conditions, including neglected or longstanding elbow fractures, recurrent dislocations, arthritis, congenital anomalies, and adhesions within the cubital tunnel. It most commonly arises as a delayed complication of elbow fractures, particularly cubitus valgus deformity resulting from the nonunion of a lateral humeral condyle fracture. The onset of ulnar neuropathy is typically due to mechanical factors such as nerve kinking, compression, and stretching, compounded by joint incongruities that disrupt normal nerve alignment and function [7].

TUNP is a chronic condition characterized by the delayed onset of ulnar neuropathy following an elbow injury. Common symptoms include vague discomfort in the medial elbow, numbness or tingling in the ring and small fingers, reduced grip and pinch strength, fatigue with repetitive hand tasks, and worsening symptoms at night or with elbow flexion, such as while talking on the phone. On physical examination, atrophy of intrinsic hand muscles, particularly the first dorsal interosseous, may be observed, along with clawing of the small and ring fingers. Wartenberg’s sign, in which the patient is unable to fully adduct the small finger, may also be present [8].

Imaging techniques such as ultrasound and MRI are essential for diagnosing schwannomas, as they help localize tumors and assess their relationship with surrounding neurovascular structures. While these modalities have limitations, they remain crucial for effective surgical planning. Ultrasound typically reveals well-defined hypoechoic masses near the nerve, whereas MRI commonly shows schwannomas as isointense on T1-weighted images and hyperintense on T2-weighted images. However, histopathological and immunohistochemical examinations remain the gold standard for diagnosis. Key diagnostic features include hypercellular (Antoni A) and hypocellular (Antoni B) regions on histopathology, along with positive S100 staining on immunohistochemistry, which differentiates schwannomas from neurofibromas [9].

In this case, HRUS failed to detect the 0.8 cm schwannoma preoperatively, likely due to its small size and deep location within the cubital tunnel. MRI was not performed, as the initial clinical impression favored isolated TUNP rather than a space-occupying lesion. The patient’s symptoms, including progressive paresthesia and weakness, were attributed to chronic traction neuropathy from cubitus valgus, without clinical or ultrasonographic evidence of a mass. However, the intraoperative discovery of the schwannoma underscores the limitations of relying solely on ultrasound for tumor detection. This case highlights a critical diagnostic challenge, while HRUS is effective in evaluating nerve compression, its sensitivity for small intraneural tumors remains limited. In retrospect, MRI may have facilitated earlier recognition of the schwannoma, potentially refining the preoperative surgical plan. Future cases of unexplained or refractory ulnar neuropathy should incorporate MRI, particularly when HRUS findings are inconclusive.

A 44-year-old male professional driver presented with a six-month history of progressive paresthesia and weakness along the medial elbow, forearm, and hand, impairing his ability to grip small objects. His symptoms were aggravated by prolonged elbow flexion, a common occupational requirement. His medical history included a childhood lateral humeral condyle fracture with nonunion, leading to cubitus valgus deformity. Physical examination revealed muscle wasting, a positive Tinel’s sign, and ulnar nerve subluxation. Electrodiagnostic studies confirmed ulnar neuropathy, while HRUS demonstrated nerve compression without a visible mass in the cubital tunnel. The patient’s cubitus valgus deformity measured 18°, which falls below the commonly recommended threshold of 20° for osteotomy. Given that the deformity had been present for 36 years without causing mechanical instability or joint pain, and that the patient had adapted functionally, osteotomy was deemed unnecessary. Additionally, supracondylar osteotomy carries risks such as loss of elbow motion, nonunion, and neurovascular injury, which outweighed the potential benefits in this case. Instead, the primary surgical goal was to relieve ulnar nerve compression, which was the immediate cause of symptoms.

During surgery, an incidental schwannoma measuring 0.8 cm was discovered within the cubital tunnel. The tumor was carefully excised, and the ulnar nerve was transposed for effective decompression. While osteotomy may still be warranted in cases of progressive deformity or recurrent neuropathy, this case highlights that anterior transposition alone can achieve excellent functional recovery in appropriately selected patients.

Nonoperative treatment is the first-line approach for mild-to-moderate cubital tunnel syndrome. However, most patients with TUNP are not suitable for conservative management due to the mechanical nature of the condition. Surgical intervention is recommended to address the local bony deformity and/or relieve pressure on the nerve [10]. While various surgical techniques have been proposed for treating post-traumatic cubitus valgus with TUNP, there is limited evidence regarding the effectiveness of surgery specifically for traumatic cubitus valgus with this condition. The success of ulnar nerve recovery following surgery depends not only on the extent of nerve involvement before the procedure but also on the correction of the cubitus valgus deformity [11].

Supracondylar osteotomy is associated with significant complications, including loss of elbow range of motion, necrosis of the lateral condyle, and persistent nonunion [12]. Therefore, the indication for osteotomy should be carefully evaluated, particularly in patients experiencing elbow pain, restricted movement, or other serious complications [13]. In patients with a carrying angle of less than 20°, osteotomy is generally avoided due to high complication risks. However, when the angle exceeds 20°, supracondylar osteotomy is recommended. For TUNP caused by cubitus valgus deformity, anterior transposition of the ulnar nerve remains the standard surgical treatment [14].

In a study by Huang et al. (2015), 62 patients with cubital tunnel syndrome underwent anterior subcutaneous transposition of the ulnar nerve. Postoperative outcomes showed that 58% had excellent results, 25% had good results, 11% had fair results, and 6% had poor outcomes. Significant improvements were noted in both DASH and VAS scores (P < 0.05), indicating substantial recovery in upper limb function and pain relief. The preoperative McGowan grade was strongly correlated with surgical outcomes, with better results seen in patients with less severe neuropathy. Age and preoperative nerve conduction velocities also influenced outcomes [15]. Similarly, a study by Thomas et al. reported significant improvements in the Dellon score (p = 0.031) and Quick-DASH score (p = 0.016) following surgery [16].

Microsurgical resection is recommended for all symptomatic schwannomas at the time of diagnosis and for asymptomatic cases with MRI evidence of tumor enlargement [17]. Complete excision is preferred initially to reduce the risk of schwannoma recurrence, emphasizing the importance of precise surgical technique [18]. Due to their eccentric and non-infiltrative growth pattern, schwannomas can often be removed with minimal damage to nerve fascicles [19]. However, despite careful dissection under magnification, neurological deficits may still occur. Takase et al. emphasized that damage to nerve fascicles during dissection is a primary cause of postoperative deficits, with the risk being higher in patients with larger tumors [20].

To determine whether similar cases have been previously reported, a structured literature search was conducted using PubMed, Scopus, and Google Scholar. The search terms included "cubital tunnel syndrome," "ulnar nerve schwannoma," "intraneural schwannoma," "lateral humeral condyle nonunion," and "post-traumatic cubitus valgus." No indexed publications were found documenting the coexistence of an occult schwannoma with cubital tunnel syndrome in a patient with lateral humeral condyle nonunion. While cases of cubital tunnel syndrome and ulnar nerve schwannomas have been described separately, no prior reports have detailed their simultaneous occurrence in this specific post-traumatic setting.

## Conclusions

This case report describes a rare instance of TUNP secondary to lateral humeral condyle nonunion, complicated by an occult ulnar nerve schwannoma in the cubital tunnel. The dual mechanisms of chronic traction neuropathy and focal compression, exacerbated by the schwannoma, were addressed through intraoperative exploration, microsurgical excision, microneurolysis, and anterior subcutaneous transposition. Despite preoperative HRUS missing the schwannoma, the patient achieved complete neurological recovery within 24 months, regaining full function and returning to work. Key insights highlight HRUS limitations in detecting small intraneural tumors, underscoring the need for advanced imaging in refractory neuropathies and intraoperative vigilance. Tumor excision was pivotal for recovery, and the case offers a surgical decision-making framework for cubitus valgus, suggesting nerve decompression may suffice without immediate osteotomy.

A literature review revealed no prior reports of cubital tunnel syndrome caused by an occult schwannoma in lateral humeral condyle nonunion, emphasizing the novelty of this presentation. Future research should investigate dual neuropathic mechanisms in post-traumatic elbow deformities and refine diagnostic protocols. MRI is recommended for unexplained ulnar neuropathy to reduce missed diagnoses and improve surgical planning. A multidisciplinary approach is crucial for addressing structural and compressive pathologies, enhancing patient outcomes.
